# Bowel Histology of CVID Patients Reveals Distinct Patterns of Mucosal Inflammation

**DOI:** 10.1007/s10875-021-01104-5

**Published:** 2021-10-01

**Authors:** Cornelia M. van Schewick, David M. Lowe, Siobhan O. Burns, Sarita Workman, Andrew Symes, David Guzman, Fernando Moreira, Jennifer Watkins, Ian Clark, Bodo Grimbacher

**Affiliations:** 1 Institute of Immunity and Transplantation, Royal Free Hospital, University College London, London, UK; 2 Institute for Immunodeficiency, Center for Chronic Immunodeficiency, Center for Translational Cell Research, Medical Center, Faculty of Medicine, Albert-Ludwigs-University of Freiburg, Breisacher Str. 115, 79106 Freiburg, Germany; 3 Pathology Department, Royal Free Hospital, London, UK; 4 Department of Pathology, Health Science Center, The University of Tennessee, 930 Madison Ave, Suite 500, Memphis, TN 38163 USA; 5 DZIF – German Center for Infection Research, Satellite Center Freiburg, Freiburg, Germany; 6 CIBSS – Centre for Integrative Biological Signalling Studies, Albert-Ludwigs University, Freiburg, Germany; 7 RESIST – Cluster of Excellence 2155 to Hanover Medical School, Satellite Center Freiburg, Freiburg, Germany

**Keywords:** CVID, diarrhea, norovirus, lymphocytes, histology, bowel

## Abstract

Diarrhea is the commonest gastrointestinal symptom in patients with common variable immunodeficiency (CVID). Different pathologies in patients’ bowel biopsies have been described and links with infections have been demonstrated. The aim of this study was to analyze the bowel histology of CVID patients in the Royal-Free-Hospital (RFH) London CVID cohort. Ninety-five bowel histology samples from 44 adult CVID patients were reviewed and grouped by histological patterns. Reasons for endoscopy and possible causative infections were recorded. Lymphocyte phenotyping results were compared between patients with different histological features. There was no distinctive feature that occurred in most diarrhea patients. Out of 44 patients (95 biopsies), 38 lacked plasma cells. In 14 of 21 patients with nodular lymphoid hyperplasia (NLH), this was the only visible pathology. In two patients, an infection with *Giardia lamblia* was associated with NLH. An IBD-like picture was seen in two patients. A coeliac-like picture was found in six patients, four of these had norovirus. NLH as well as inflammation often occurred as single features. There was no difference in blood lymphocyte phenotyping results comparing groups of histological features. We suggest that bowel histology in CVID patients with abdominal symptoms falls into three major histological patterns: (i) a coeliac-like histology, (ii) IBD-like changes, and (iii) NLH. Most patients, but remarkably not all, lacked plasma cells. CVID patients with diarrhea may have an altered bowel histology due to poorly understood and likely diverse immune-mediated mechanisms, occasionally driven by infections.

**Supplementary Information:**

The online version contains supplementary material available at 10.1007/s10875-021-01104-5.

## Introduction

Common variable immunodeficiency (CVID) is the commonest symptomatic primary immunodeficiency in humans [1] with a wide spectrum of clinical complications [2]. Patients have very low immunoglobulin levels of IgG and IgA or IgM with a poor vaccination response due to impaired B-cell function, a predisposition to infection, and autoimmune or granulomatous manifestations [3]. Up to 47% of investigated CVID patients suffer from abdominal symptoms [4–8].

Studies on the histopathological correlates of CVID-related abdominal symptoms have shown a variety of findings in the small intestine. Commonly reported are lymphoid hyperplasia (LH), villous atrophy, raised numbers of intraepithelial lymphocytes (IEL), duodenitis, graft versus host disease-like changes, or no histopathological abnormalities. In contrast to non-CVID patients, most CVID patients lack plasma cells in the lamina propria, even when there is inflammation in the bowel [8–14]. Herbst et al. found that a total absence of IgA_1+2_ and IgG_2_ producing plasma cells occurred very often in CVID patients and that a defect of intestinal immunoglobulin producing plasma cells correlates with lower levels of the respective isotype in the peripheral blood [10].

Features of inflammatory bowel disease (ulcerative colitis, Crohn’s disease) and microscopic colitis can be found in bowel biopsies of CVID patients. These may include lymphoid hyperplasia, granulomas, ulcers, crypt distortion, and graft versus host-like lesions. The main difference from classical inflammatory bowel disease is that plasma cells are either rare or absent in biopsies from CVID patients [8, 12, 13].

Nodular lymphoid hyperplasia (NLH) of the small and large bowel is seen frequently in biopsies from CVID patients. It is defined as an accumulation of nodules of at least 1 mm in diameter [15] with a germinal center [16]. Lymphocytic colitis, in contrast, is characterized by an increase in intraepithelial lymphocytes without germinal center formation [17]. In immunocompetent adults, nodular lymphoid hyperplasia is found only rarely and usually in association with infection [18, 19] but in patients with immunodeficiencies nodular lymphoid hyperplasia is frequent [20, 21]. The etiopathology of nodular lymphoid hyperplasia is unclear. While it is frequently found in CVID patients who have abdominal symptoms [14, 15], it is also sometimes identified in patients without abdominal complaints [16, 22] and might be a sign of immune dysregulation rather than a primary pathological injury.

The number of eosinophil granulocytes in the gastrointestinal tract mucosa of healthy individuals increases significantly from the esophagus to the ileum with up to 74.38 ± 41.77 eosinophils per ileal mm^2^ and then decreases again towards the left colon [23]. Parasitic infections, inflammatory bowel disease, vasculitis, drug reactions, e.g., to NSAIDs, malignancies, and primary eosinophilic enteritis can be reasons for an increase of eosinophils in the intestinal tissue [24, 25]. Precise quantification of eosinophils and cut-off values for eosinophilia in the different pathologies named above have unfortunately not yet been established [24]. In primary eosinophilic gastroenteritis, an excess of eosinophils would be expected, as well as mucosal oedema, mucosal hyperaemia [26–28], and ulcerations [29, 30].

Some clinicians use the term CVID enteropathy for small bowel, “biopsy-proven lymphocytic infiltration in the lamina propria and interepithelial mucous with villous atrophy, insensitive to gluten withdrawal” [31]. This entity will further be referred to as coeliac-like enteropathy. Histologically, it resembles coeliac disease but a gluten-free diet is rarely of help in CVID patients, HLA typing often excludes coeliac disease and coeliac disease autoantibodies are detected only infrequently in CVID patients [14, 32].

The existing studies on the histopathological picture in patients with CVID-associated abdominal symptoms have demonstrated that findings vary widely. Often, particular features are identified in CVID patients which are inconsistent with classical IBD or coeliac disease. Frequently, the histopathological findings may not provide a sufficient explanation for the symptoms experienced by the patient [22]. For these reasons, there is a suggestion that CVID bowel disease is a separate entity and does not fit common classifications [9].

Gastrointestinal infections often occur in immunodeficient patients [8, 33]. A small study suggested that the histopathological picture of coeliac-like enteropathy in CVID patients may be associated with chronic norovirus infection [34, 35]. Brown et al. suggested that in norovirus*-*infected patients, similar cytotoxic mechanisms underlie the villous atrophy in coeliac disease and norovirus infection [36]. Polymerase chain reaction (PCR) is used to diagnose norovirus infections [37]. *Giardia lamblia* is a common infective cause for acute and chronic diarrhea in CVID patients [8, 38] and can be challenging to eradicate [39]. It has often been found in association with nodular lymphoid hyperplasia [20, 40] and can also cause villous atrophy in the small bowel. Detection of *Giardia lamblia* in the stool is more sensitive via PCR than through microscopy [41]. Cytomegalovirus has long been identified as a causative organism of colitis, predominantly in immunocompromised patients [42, 43] and as a factor complicating inflammatory bowel disease [44]. Furthermore, CMV-related ileitis has been described in immunocompromised patients, involving ulceration, gastrointestinal bleeding, and diarrhea [45–47], which respond to virostatic treatment [48, 49].

The aim of this study was to describe the histological picture of CVID patients’ gut mucosa, especially of those suffering from diarrhea, and correlate this with gastrointestinal infection and lymphocyte phenotyping, in the Royal-Free-Hospital (RFH) London CVID cohort.

## Materials and Methods

We performed a retrospective study of adult CVID patients. Forty-four out of 172 patients diagnosed with CVID at our Primary Immunodeficiency Center were included.

Available histopathological samples from the bowel were retrieved and reassessed following a standardized proforma. Only these newly assessed biopsies were used; endoscopy reports without biopsy and histology reports without available histology slides were not included in the study. Endoscopy reports and clinic letters were checked for information on the presence of an infection at the time of biopsy. For some patients, infection with norovirus was detected later by PCR of the stool. Further recorded infections were based on tests performed at local diagnostic laboratories (gastroenteritis multiplex polymerase chain reaction (PCR) including PCR for noro-, adeno-, sapo-, rota-, and astrovirus, multiplex PCR for *Giardia intestinalis*, *Cryptosporidium species*, *Entamoeba histolytica*, *Microsporidium species*, microscopy, culture, and sensitivity, cryptosporidium microscopy, PCR for Salmonella, verotoxigenic *Escherichia coli*, *Shigella*, *Campylobacter*, Ova, Cyst, and parasite microscopy, *Clostridium Difficile* glutamate dehydrogenase test).

Recordings of lymphocyte phenotyping were reviewed. Measurements of cells with the following markers from the earliest and most recent phenotyping in the clinic were analyzed and correlated with histological features: absolute numbers of CD3 + , CD4 + , CD8 + , CD16/56 + , CD19 + , and CD19 + switched memory cells (CD19 + CD27 + IgD/M-). Absolute lymphocyte number, percentage of CD4 + cells, and percentage of naïve CD4 + cells (% of CD4 + CD45RA +) was used to calculate absolute numbers of naïve CD4 + cells.

Biopsies obtained during esophagogastroduodenoscopy were of duodenal origin, and biopsies taken during colonoscopies were either colonic or ileal. By revisiting the histological reports and clinic letters from the time of endoscopy, we inferred the indications for the endoscopy and the symptoms present. As not all patients had their biopsies taken at the Royal Free Hospital, the slides of three patients’ biopsies were obtained from their local hospital.

Features assessed in biopsies from all sites were (i) lymphoid hyperplasia and (ii) presence of plasma cells. For colonic biopsies, features were (i) cryptitis (= two or more granulocytes/crypt-diameter), crypt abscesses, crypt distortion/increased inflammatory cells in the lamina propria (inflammatory bowel disease pattern, with or without acute component), (ii) increased subepithelial collagen (collagenous colitis pattern) or lymphocytic colitis (pure intraepithelial lymphocytosis), and (iii) pure acute colitis. For duodenal/ileal biopsies, patterns were (i) flattened villi, (ii) increased intraepithelial lymphocytes (if i and ii present, coeliac-like pattern), (iii) cryptitis (= two or more granulocytes/crypt-diameter), and (iv) acute inflammation, acute duodenitis/ileitis.

We define acute duodenitis, ileitis, or colitis as a pattern of injury characterized by neutrophilic inflammation in the mucosa. If in addition to the lamina propria, neutrophils entered the crypt or surface epithelium, then the term acute cryptitis or surface epitheliitis is used as a correlate of epithelial damage. If the inflammation is more severe, crypt abscesses may form, where neutrophilic inflammation lies free as exudate within crypt lumina. Acute enteritis is most commonly seen in bacterial infection or in exacerbations of inflammatory bowel disease. There is no fundamental distortion of underlying colonic crypt architecture.

Crypt architectural injury arises as a result of prolonged mucosal injury, as in inflammatory bowel disease or chronic colitis of other causes. The defining histological feature of chronic injury is crypt architectural distortion, wherein crypts become shortened, branched, or destroyed. There may be Paneth cell metaplasia of crypt cells. If there is chronic active colitis, the changes of acute colitis may be superimposed on architectural changes. Granulomatous inflammation is a feature of chronic injury, and may be seen in Crohn’s disease, in addition to other architectural changes of chronicity.

By contrast, intraepithelial lymphocytosis in gut mucosa implies an immunological or, less commonly, viral etiology. In the duodenum, it is frequently accompanied by villous atrophy to give rise to the characteristic changes of coeliac disease. In the colon, lymphocytic infiltration of surface and crypt epithelium without alteration of mucosal architecture is a diagnostic feature of lymphocytic colitis in immunocompetent patients.

CVID patients provided written informed consent under study protocols approved by NHS Research Ethics Committees (REC 04/Q0501/119).

## Results

Ninety-five histology samples from 44 patients were reviewed. Many patients had had several endoscopies and so metachronous sets of biopsies from the same part of the gut were available. Biopsy results were summarized in a cumulative report including duodenum, ileum, or colon for each patient (Table 1). Thirty-five patients underwent endoscopy because they were suffering from diarrhea or loose stools. Two of these had had diarrhea, but at the time of endoscopy, this had settled. Further reasons for endoscopy were other abdominal symptoms such as “altered bowel habits” (unspecified), dyspepsia or abdominal pain as well as malabsorption, anemia, suspected gastrointestinal infection, suspected protein-losing enteropathy, suspected coeliac disease, and positive fecal occult blood (Table 1).

**Table 1:** Histopathology in colon, duodenum, and ileum. *PCs* plasma cells, *IEL* raised intraepithelial lymphocytes, graded + to +  +  + , *NLH* nodular lymphoid hyperplasia, *LH* lymphoid hyperplasia (no/small (< 1 mm) germinal centers);—no biopsy available. Only pathological patterns described. * infected patient; *N* norovirus, *G* Giardia lamblia, *CMV* cytomegalovirus, *C* Campylobacter. The column “reason” gives the reason for endoscopy if available: *D* diarrhea, *M* malabsorption, *A* anemia, *altBh* altered bowel habits, (*D*) diarrhea was the reason for endoscopy, but it had settled at biopsy. *i* infection suspected, *prot.* protein losing enteropathy suspected, *c* coeliac disease suspected, *dysp.* dyspepsia, *P* abdominal pain, *FOB* fecal occult blood, *wl* weight loss, *v* vomiting

Patient	Colon	Duodenum	Ileum	Reason
*1*	Acute cryptitis, no PCs	-	-	D, i
*2*	Normal PCs	-	-	D
*3*	Normal PCs	-	-	D
4*N	No PCs	Villous atrophy, no IEL, cryptitis, NLH, no PCs	-	(D), wl, i
*5*	Acute cryptitis, distortion, abscesses, NLH, no PCs	Villous atrophy, no IEL, acute duodenitis, cryptitis, no PCs	-	D, c
*6*	Acute cryptitis, raised IEL(lymphocytic colitis), no PCs	No PCs	-	D
*7*	Acute cryptitis, distortion, abscesses, no PCs	No PCs	-	Wl, dysp
8*N	Acute cryptitis, abscesses, NLH, no PCs	Villous atrophy, IEL + + , NLH, no PCs	-	D, A
*9*	Acute cryptitis, (N)LH, few PCs	NLH, few PCs	-	D
*10*	Few PCs	Normal PCs	-	(D)
*11*	NLH, few PCs	Few PCs	-	D
*12*	NLH, no PCs, patchy eosinophilia	NLH, no PCs	-	D
*13*	NLH, no PCs	NLH, no PCs	-	D, dysp
*14*	No PCs	NLH, no PCs	-	D
*15*	Normal PCs	Normal PCs	-	D, i, dysp
*16*	Normal PCs	Normal PCs	-	D, M, dysp., prot
17 *N	Acute cryptitis, NLH, no PCs	Villous atrophy, IEL + + + , no PCs	-	D, wl
18 *N	No PCs	Villous atrophy, IEL + + , no PCs	Villous atrophy, IEL + + + , no PCs	D
*19*	LH, few PCs	LH, few PCs	No PCs	D, dysp., P
*20*	Acute cryptitis, eosinophilia, NLH, no PCs	No PCs	Acute + chronic ileitis, crypt abscesses, polymorphs surf. Epithelium, mild eosinophilia, villous atrophy, no IEL, no PCs	D, FOB
*21*	NLH, few/no PCs	Few PCs	NLH, few PCs	D
22 *CMV	Acute cryptitis, abscesses, no PCs	No PCs	Acute ileitis, ulcers, no PCs	D, A, wl, prot
*23*	Acute cryptitis, distortion, no PCs	Acute duodenitis, no PCs	Acute ileitis, no PCs	D
*24*	Crypt abscesses, no PCs	No PCs	No PCs	n/a
*25*	NLH, no PCs	NLH, normal PCs	LH, few PCs	D
*26*	NLH, no PCs	Cryptitis, no PCs	LH, no PCs	D
*27*	NLH, few PCs	Normal PCs	NLH, normal PCs	D, wl, M
28 *N	No PCs	No PCs	No PCs	D, wl, dysp., M
29 *G	-	NLH, no PCs	-	D, i
*30*	-	NLH, no PCs	-	D, i
31 *N	-	Villous atrophy, IEL + + , infl. in lamina prop, no PCs	-	D, wl
*32*	-	Villous atrophy, IEL + + , no PCs	-	Dysp., reflux
*33*	-	Villous atrophy, IEL + + + , normal PCs	-	A, D
*34*	-	Villous atrophy, no IEL, acute duodenitis, cryptitis, crypt abscesses, focal eosinophilia, no PCs	-	D
*35*	-	Few PCs	-	D
*36*	NLH, no PCs	NLH, no PCs	-	D
*37*	-	NLH, no PCs	-	Reflux, gastritis
*38*	-	No PCs	-	wl, iron def., 2^nd^ look after ulcers
*39*	-	No PCs	-	D, M
*40*	-	No PCs	-	M, v
*41*	-	No PCs, IEL +		M, i
*42*	-	Normal PCs		M, c
*43*	-	Normal PCs	NLH, no PCs	D
44 *G,C	-	No PCs	NLH, no PCs	Check response to chemotherapy after gastric lymphoma and C. diff

Forty-six duodenal samples from 41 patients were reviewed. Samples from 19 patients (46.3%) did not show any abnormalities.

Duodenal samples from 29 patients (70.7%) showed no plasma cells at all, four patients (9.8%) had severely reduced numbers, and eight patients (19.5%) had a normal number of plasma cells, six of these without any histopathological abnormalities.

Lymphoid hyperplasia was seen in twelve patients’ duodenal samples (29.3%); eleven of them had germinal centers of at least 1 mm diameter.

Villous blunting was seen in nine patients (22%) of whom six also had raised intraepithelial lymphocytes (66.6%). Increased intraepithelial lymphocytes was subdivided into three categories: mild (one patient, #41), moderate (four patients, 57.2%), and severe (two patients, 28.6%, #17, 33). One patient with villous atrophy had normal numbers of plasma cells (#33); one patient had villous atrophy, raised IEL, and accompanying inflammation in the lamina propria (#31). One further patient (#41) had a mild and patchy increase in IEL but no villous blunting.

**Fig. 1:**
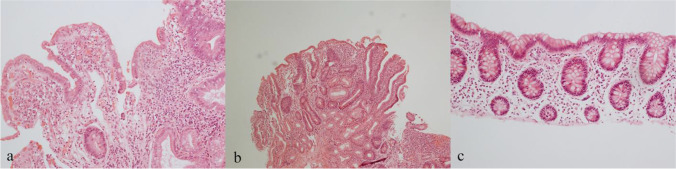
**a** and **b** Acute duodenitis and villous atrophy, not typical of coeliac disease. **c** Normal colon

Acute duodenitis was seen in three patients (7.3%), of whom two also had villous atrophy but no raised IEL (Fig. 1a and b). Crypt abscesses were seen in one of these patients who also had eosinophilia (#34, Table 1). Four patients had cryptitis (9.8%).

*Giardia* organisms were seen in the duodenal biopsy of patient #44.

Fourteen ileal samples from 13 patients were reviewed. Samples from ten patients (76.9%) had no plasma cells, two (15.4%) had few plasma cells, and one had a normal amount of plasma cells (7.7%). Three of those with no plasma cells had no other pathological features in the ileum (30%).

Lymphoid hyperplasia was seen in six patients’ ileal samples (46.2%); two of them had germinal centers of at least 1 mm diameter. Villous atrophy was seen in two patients (15.4%) of whom one had raised IEL (#18) and one had ileitis (#20). Three patients (23.1%) had acute ileitis in total. One of these also had chronic ileitis, crypt abscesses, polymorphs in the surface epithelium, mild eosinophilia, and villous flattening (#20). One other patient with acute ileitis had ulcers and very rare CMV inclusions (#22).

Thirty-five sets of colon biopsies from 29 patients were reviewed. Samples from 19 patients (65.5%) had no plasma cells at all, six (20.7%) had few, and four (13.8%) had a normal number of plasma cells. Samples from four patients (13.8%) did not have any abnormalities, and samples from five patients (17.2%, Fig. 1c) had only a lack of plasma cells (few or no plasma cells present).

Lymphoid hyperplasia was seen in 14 patients (48.3%), 13 of them had germinal centers of at least 1 mm diameter, and one patient had smaller germinal centers.

Crypt abscesses were seen in five patients (17.2%). Cryptitis was seen in ten patients (34.5%), four of these had crypt abscesses (40%), three had crypt distortion (30%), and two had eosinophilia (#12, 20). Two patients had a histological pattern like inflammatory bowel disease with cryptitis, crypt abscesses, and distortion (#5, 7) but lacked plasma cells. One of these also had NLH (#5). One patient presented with lymphocytic colitis but lacked plasma cells (#6). In four patients’ biopsies, colonic inflammation was either graded as moderate (#1) or mild acute colitis (#20, 22, 23). The main findings are summarized in Fig. 4.

### Infection as Cause of Diarrhea in CVID

An infection was identified in nine of the 44 patients (20.45%), eight of whom had diarrhea. For the remaining 35 patients, no infectious agent was either visible in the biopsied specimens, or identified by conventional microbiological testing at the time of biopsy.

Three patients were known to have an infection prior to or at the time of biopsy: two with *Giardia lamblia*, one of these additionally had recurrent infections with *Campylobacter jejunii* (#44), and one with norovirus. In one patient, CMV inclusion bodies were found in the ileal biopsy. Five additional patients were found to be positive for norovirus after the biopsy had been taken (Table 2). By calculating divergence and ancestor dates [35], the infection interval was estimated and it was assumed that the infection had been present at the time of biopsy in patients #4, 8, 17, 18, and 31.

**Table 2:** Gastrointestinal infections detected during medical care at the RFH

Patient	Before biopsy	After biopsy
*4*		C. diff pos. (but toxin neg) 4 years after sampling. Norovirus (first positive 6 years after sampling)
*5*		Campylobacter infection 2 years after duodenal sample and 7 years after colonic sample (4 × pos. Over 3 months)
*7*	Multiple pos. CMV PCR in the blood	
*8*	Campylobacter infections 4–6 years before colonic sample	Campylobacter infections 1, 5 and 8 years after duodenal sample. First norovirus positive 7 years after duodenal sample
*10*	Salmonella positive once 6 years before sampling	
*11*		Norovirus positive 1 year after sampling (once only)
*17*		Norovirus first positive same year
*18*		Norovirus positive first time 1 year after last small bowel sample
*22*		Campylobacter infection 3 years after last sample
*23*		C. difficile toxin positive 4 years after sampling
*24*	Campylobacter infection 7 months before sampling	Campylobacter infections 2, 3, and 6 years after sampling
*28*	*H. pylori* 8 years before sampling	Norovirus
*29*		Giardia positive 6x, 9–16 years after sampling
*31*	Norovirus first pos. 4 months before sampling	Norovirus. C. diff toxin pos. 1 1/2 years after; C. diff pos. (toxin neg) 2 years after sampling
*32*		H. pylori positive 2 months after sampling (5 × positive over 1.5 years)
*33*	Campylobacter infection 2 years before sampling	
*38*	Campylobacter infection 9 years before sampling	
*41*		H. pylori positive 2, 3, and 4 years after sampling
*43*	*H. pylori* 7 years before sampling	Salmonella 1 year after sampling (4xpositive)
*44*	Campylobacter 5 and 1 year before duodenal sample. Giardia positive 5 years and again 6 months before duodenal sample	Campylobacter and Giardia 4 months after ileal sample, again 6 months and 1 year (Campylobacter) and 6 and 7 years after biopsy (both)

**Fig. 2:**
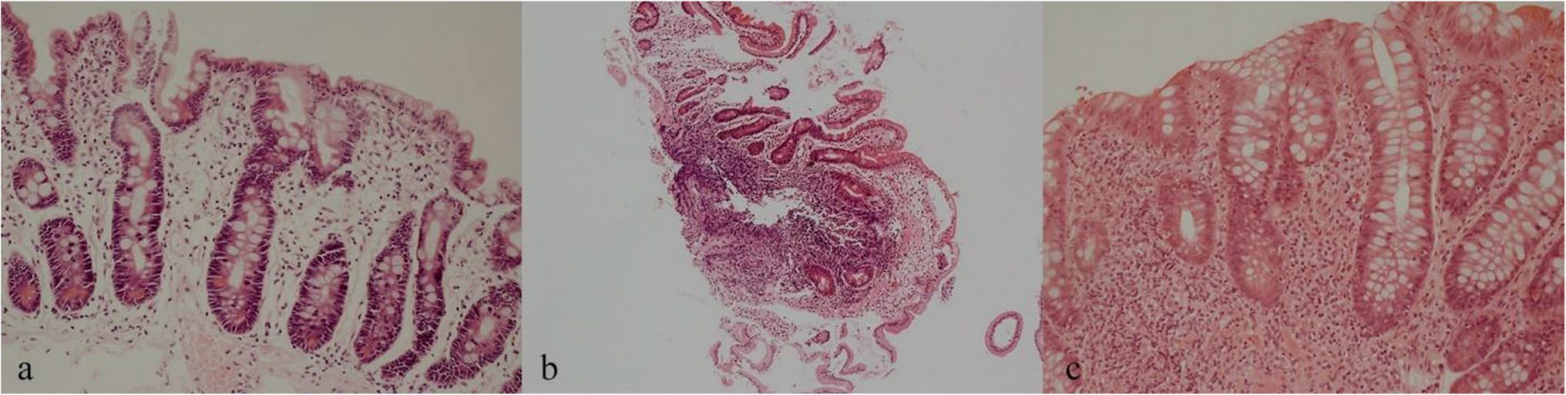
**a** Duodenum with villous atrophy, increased intraepithelial lymphocytes (coeliac-like changes), and no plasma cells. **b** Duodenum with normal architecture, no plasma cells, and a lymphoid follicle. **c** Rectum with mild crypt distortion, cryptitis, and mild loss of chronic inflammatory cell gradient mimicking IBD but with paucity of plasma cells. ©Dr. Jennifer Watkins, Royal Free London, Department of Cellular Pathology

Four out of six patients with norovirus had duodenal villous atrophy with raised IEL, regarded conventionally as a coeliac-like picture (#8, 17, 18, 31; Table 1, Fig. 2). Patient 18 additionally had a coeliac-like histology in the ileum. Patient 17 had acute colonic cryptitis and NLH as well as patient 8, who also had colonic abscesses. Patient 31 had a coeliac-like histology with an inflammatory infiltrate in the lamina propria. Patient 4 was norovirus positive and had villous atrophy without raised IEL but with cryptitis and NLH. Patient 28 did not show any abnormalities except for an absence of plasma cells.

**Fig. 3:**
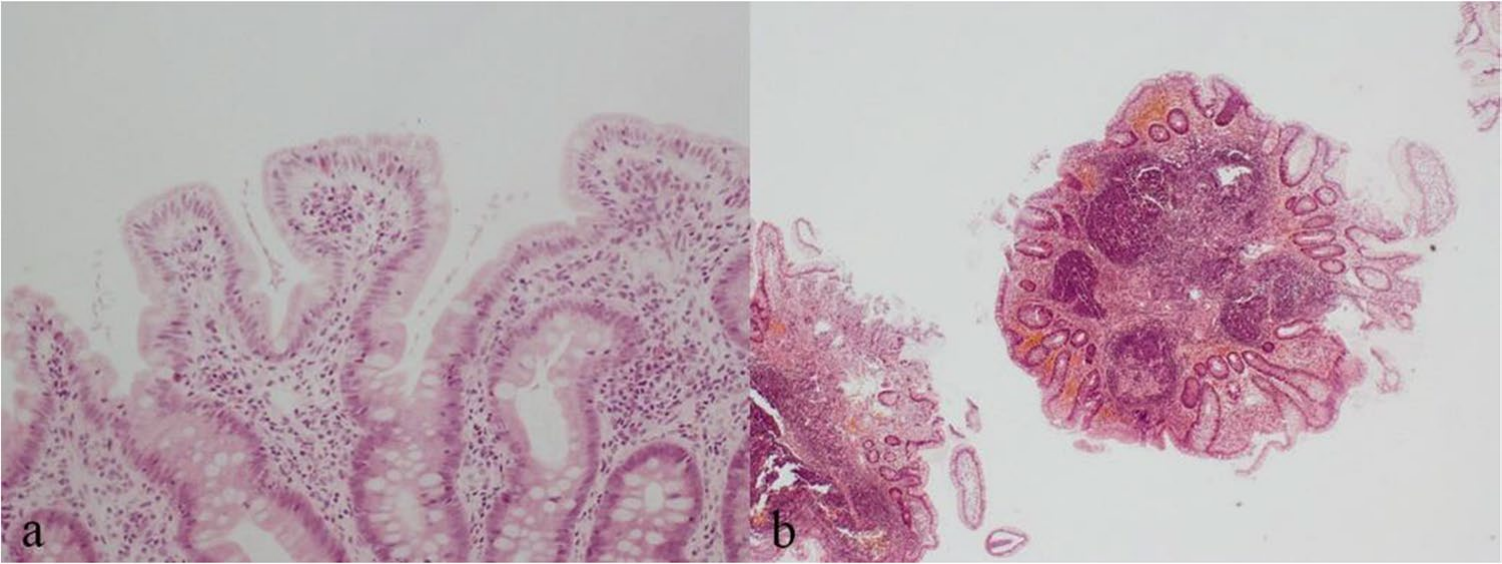
**a** Duodenum with Giardia and (**b**) with florid nodular lymphoid hyperplasia in the same patient

Both patients infected with *Giardia lamblia* had nodular lymphoid hyperplasia only (#29 and 44, Fig. 3).

The patient with CMV inclusion bodies in the ileum had severe ileitis with eosinophilia (#22).

Table 2 lists all relevant detected GI-infections in the presented cohort that were found during the time of medical care at the RFH since 2002. It is striking that many patients suffer from recurrent *Campylobacter jejunii* infections, though this was in most cases not close to the time of sampling.

### Auto-inflammation as Cause for Diarrhea in CVID

Inflammation such as ileitis, duodenitis, colitis, or features of cryptitis or crypt abscesses were seen in eight patients (29.6% of diarrhea patients without infection), and in three patients with other symptoms and without infection. Lymphocytic colitis was present in one patient. Granulomas were not seen in any samples. Two patients had an IBD-like pattern with a lack of plasma cells (#5 and 7, Fig. 2c).

Eosinophils were increased (defined as > 10 eosinophils per high power field) in biopsies of three non-infected patients. One patient had focal eosinophilia in the duodenum (#34) associated with acute inflammation (acute duodenitis with villous atrophy, cryptitis, and crypt abscesses). The other two patients had eosinophilia in the terminal ileum and colon together with colitis and ileitis, but not in the duodenum (#20), or patchy eosinophilia in the colon together with NLH (#12). The degree of eosinophilia in these colonic biopsies might be explained by the biopsies originating from the right colon. Eosinophils have been described to be increased in the right colon, with a decrease towards the left colon [23]. Unfortunately, the exact origin of the colonic biopsies could not be determined.

Only two patients (#32 and 33) had a coeliac-like picture in duodenal biopsies without having a known infection, one of them suffered from diarrhea, while the other one had dyspepsia and reflux symptoms at the time. Both patients’ symptoms had resolved at follow-up without having received any specific therapy and we cannot exclude the possibility that they had infection at the time of biopsy.

### Lymphoid Hyperplasia

Twelve patients without infection (34.3%) had (nodular) lymphoid hyperplasia (Fig. 2b) as the only histopathological feature; eleven of these had diarrhea.

### Plasma Cells

Most patients lacked plasma cells in some sampled part of the bowel. Six patients (13.6%) had normal numbers of plasma cells in the lamina propria of the sampled GI-tract and no detected area with a lack of plasma cells. In four of these cases, biopsies were only taken in one gastrointestinal area. Five of the six did not have any histopathological abnormalities (83.3%), of whom four had diarrhea. One further patient with diarrhea presented with a coeliac-like picture and normal PCs in the duodenum (#33).

### Search for Biomarkers of “CVID Enteropathy”

We looked for correlations between lymphocyte numbers and the most frequent histological features in all 44 biopsied patients. However, no significant differences or associations were detected between groups using the categories (i) coeliac-like histology, (ii) IBD-like, or (iii) NLH-only. We also compared lymphocyte phenotyping results between patients with any signs of colitis (including IBD-like colitis, acute colitis, and lymphocytic colitis) and no colitis, as well as between those with raised IEL and normal IEL and normal numbers of plasma cells versus no/few plasma cells in the lamina propria. We further compared those with and without inflammatory changes (other than coeliac-like as main feature) and those with/without normal mucosa. No significant differences were detected in any of these analyses by the Mann–Whitney *U* test (see Supplementary material for details).

### Genetics

In 14 out of 24 patients who were sequenced in this cohort, a genetic mutation was identified (Table 3). One patient (# 24) had a mutation in the *IKZF1* which encodes the transcription factor IKAROS. This patient had histological colonic inflammation and, not at the time of biopsy, several infections with *Campylobacter*. Of the two patients who had a pathogenic mutation in *CTLA4*, one had an IBD-like colitis (# 7); the other one had a lymphocytic-colitis-like histology (# 6). Two patients had pathogenic variants in *NFKB1*, of whom one had a coeliac-like inflammation in the small bowel but was additionally infected with norovirus (# 18); the other patient had an acute duodenitis and ileitis, as well as signs of colitis. One further patient had a mutation in *NFKB2*; his histology revealed NLH in colon and duodenum (# 36). Four patients had mutations in *TNFRSF13B* (het(A181E), het(C104R), het(G278S)), encoding for the transmembrane activator and CAML interactor (TACI); all four had gastrointestinal NLH. One patient with colonic inflammation, coeliac-like duodenitis, and NLH, who was infected with norovirus and had recurrent episodes of *Campylobacter* infections, was found to have a loss-of-function mutation in *PTPN2* (# 8). One patient without histopathological abnormalities had a novel frameshift variant in *SOCS1* (# 10). Two patients (# 11 and 39) were diagnosed with an activated-PI3K-delta-syndrome (APDS) manifesting as CVID, one with a mutation in *PIK3CD* (APDS1), and one with a mutation in *PIK3R1* (APDS2). The patient with the *PIK3CD* mutation had NLH and was positive for norovirus in a stool sample once (#11).

**Table 3:** Genetic analysis was performed in 24 out of 44 patients; in 14 of them, a genetic diagnosis was established. This table lists the defect genes and which patients are affected

*Genetic analysis performed*		*24/44*
*Genetic diagnosis made*		14/24
*Mutations in CTLA4*		# 6, 7
*NFKB1*		# 18, 23
*NFKB2*		# 36
*PTPN2*		# 8
*PIK3CD*		# 11
*PIK3R1*		# 39
*SOCS1*		# 10
*IKZF1*		# 24
*TNFRSF13B*	A181E, C104R, G278S	#21; #30, 43; #37

## Discussion

A high prevalence of coeliac-like enteropathy and signs of inflammation has been reported as a common finding in CVID patients [9, 13, 32]. The prevalence of increased IEL and villous atrophy was lower in our RFH-CVID cohort compared to other studies which themselves show wide variations: 63% of 19 patients (with no regard to symptoms) had increased IEL and 52.6% additionally had villous blunting in the small bowel in a study by Daniels et al. 13; 51.2% of 41 CVID patients with GI symptoms had villous blunting and 75.6% increased IEL in a study by Malamut et al. [14]. Villous blunting was seen in 31.2% of 32 CVID patients with anemia or GI symptoms in a prospective study by Luzi et al. [50], and in 33% of 17 mostly symptomatic CVID patients by Herbst et al. [10]. Villous atrophy was seen in 50% of 14 CVID patients by Mannon et al. [51] comparing patients with and without GI symptoms. Raised IEL were present in 64.3%. This compares to 17.1% of patients with small bowel biopsies with increased IEL and 24.4% with villous atrophy in our study.

The role of eosinophils has not yet been studied in the context of gut disease in CVID. Biagi et al. have described one biopsy with an inflammatory infiltrate of mainly eosinophils infiltrating duodenal mucosa without further discussion [32]. Malamut et al. listed eosinophils as part of the inflammatory infiltrate in CVID patients with duodenitis in several cases [14]. Three of our patients had mild eosinophilia, two in colonic/ileal biopsies that would suggest origin in the right colon or terminal ileum which can be assumed to be physiological [23]. The remaining patient had focal eosinophilia in the duodenum associated with acute inflammation, which we interpreted as being part of the inflammatory process [52].

In colonic biopsies, the study by Teahon recorded many more patients with microscopic colitis [9] whereas we had only one patient presenting with features of lymphocytic colitis. Also, granulomas were not observed in any biopsy which was the case in most other similar studies.

Daniels et al. had far more patients with lymphoid aggregates in their colonic biopsies (81%), though our proportion (47.8%) is comparable to that of Maarschalk et al., who found nodular lymphoid hyperplasia in 53% of biopsies [22], of Herbst et al. with 41.2% nodular lymphoid hyperplasia in their small bowel biopsies, and of Oksenhendler et al. who found nodular lymphoid hyperplasia in 55.9% of CVID patients with diarrhea [8, 10].

**Fig. 4:**
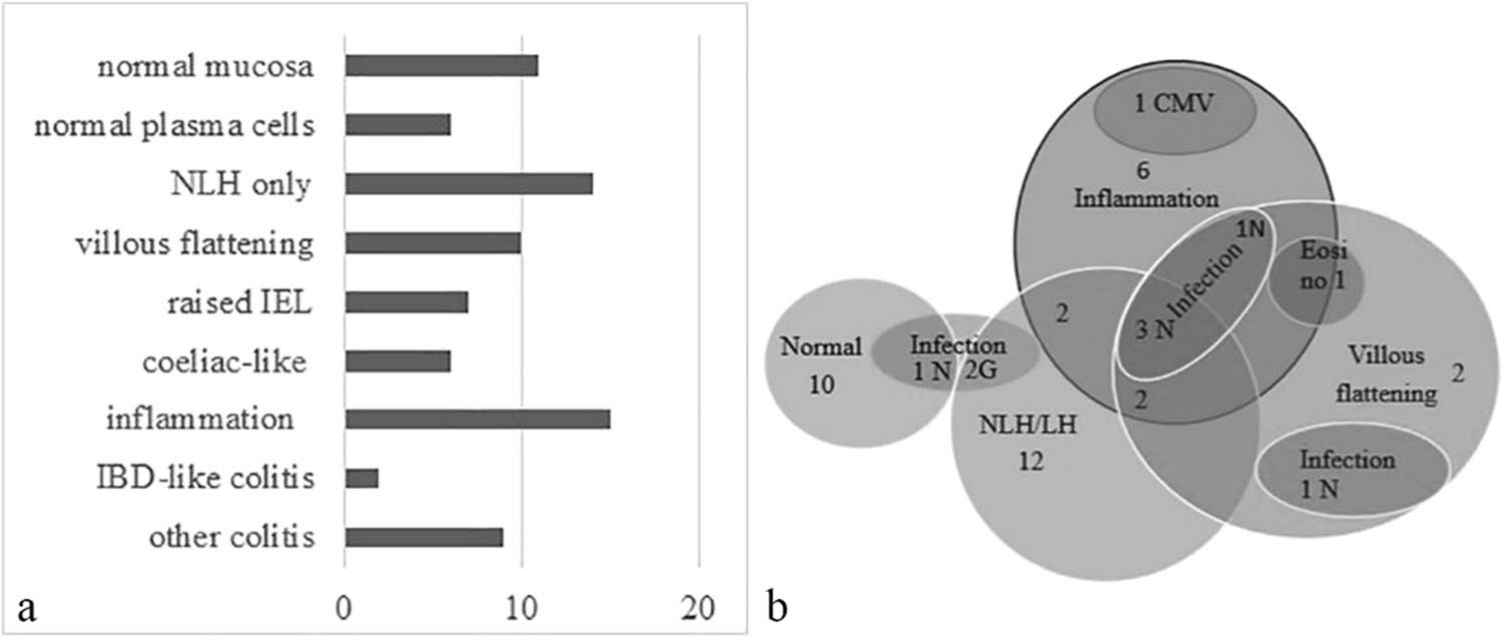
**a** Histological features. **b** Histological features and infection, all 44 patients included. The group “Inflammation” includes one patient with raised IEL only. N= norovirus, G= Giardia lamblia, CMV=cytomegalovirus

Figure 4b shows that NLH as well as inflammation often occur as single features whereas villous flattening predominantly occurs together with other features. Infection, taken as a single group including viruses and parasites, did not have a clear association. However, this may be influenced by sampling: for example, the patient with chronic norovirus infection but “normal” duodenal histology had evidence of villous blunting in other parts of the small bowel on capsule endoscopy.

Plasma cells are usually abundant in the lamina propria of GI tract mucosa, aside from the esophagus and stomach. In those patients where they were absent, the lamina propria was edematous and “empty-looking.”. In cases where we assessed plasma cells as being decreased in number, plasma cells were identified only after diligent search. In a number of patients, plasma cells were present in the upper GI tract biopsies, but absent in ileal or colonic biopsies, but not the other way around. Daniels et al. as well as Biagi et al. suggested that a lack of plasma cells in GI biopsies implies CVID but that CVID cannot be excluded if there are plasma cells present [13, 32]. Absence of plasma cells is not specific to CVID patients and can also occur in patients with other primary immunodeficiencies such as X-linked agammaglobulinemia or Ataxia-telangiectasia [33]. Jorgensen et al. found a paucity of plasma cells “to be associated with signs of increased systemic inflammation (i.e., increased serum levels of sCD14 and sCD163) and immune activation (sCD25)” [53]. These were not analyzed in our cohort, where 13.6% of the patients had identifiable plasma cells in their biopsies. It was interesting to find that two patients had absent/very few plasma cells in the colon, but normal plasma cells in the duodenum.

Our incidence of a coeliac-like pattern with simultaneous norovirus infection corresponds more with the results of Jorgensen et al. [53] than with those of Woodward et al. [34] (four out of six versus eight out of eight). Two patients without proven infection had a coeliac-like picture but no symptoms at follow-up and one of these had no villous atrophy in a follow-up capsule. We therefore assumed that in these two cases, an undiagnosed infection might have been the reason for the described histological changes and symptoms at the time of biopsy. PCR of the reviewed samples would be needed to give an exact number of those patients who were norovirus positive at the time of sampling. Both patients with recurrent *Giardia* infections had lymphoid hyperplasia as described by Webster et al. and Pérez-Roldan [20, 54].

One patient (#22) had CMV inclusion bodies in her ileal biopsy, along with ulcers. At the time of biopsy, this was interpreted as of questionable relevance and the patient was not treated with antiviral drugs. Daniels et al. also reported on one patient with CMV in the large and small bowel [13]. There are case reports where a positive effect of virostatic therapy on patients’ symptoms has been described [48, 49]. However, no larger studies exist that look at ileal histology in apparent CMV-related ileitis before and after antiviral treatment as an ileal CMV infection (not pouchitis) is rare. CMV PCR of bowel biopsies and CMV PCR of the blood of patients with diarrhea should be performed in CVID patients to advance our knowledge in this field [55].

For norovirus, the critical question must be asked whether this is causative of (coeliac-like) enteropathy or whether infective agents are only an exacerbating factor in a pre-existing enteropathy as has been observed in non-CVID IBD patients [56]. Gathmann et al. hypothesized that enteropathy might “represent an ineffective or inappropriate gastrointestinal response to subclinical infection” [57]. For nodular lymphoid hyperplasia, Webster suggested a similar explanation many years ago [20] and it has also been postulated for other histologies and infective agents [14, 33, 51]. However, the mucosal appearance in chronic norovirus infection is the same in other immunosuppressed patients without CVID [35] and resolves with clearance of the virus, suggesting that this is more likely to be a reaction to the virus. A susceptibility towards gastrointestinal infections in CVID patients has been ascribed to the lack of intestinal IgA [10, 58], but most patients with chronic norovirus infection also have impaired T cell function [35]. No significant differences in lymphocyte populations could be found in the current study comparing patients grouped by histopathological features. Among others, Agarwal et al. suggested a T-cell defect to “account for mucosal dysregulation” in CVID [59]; our results do not support or contradict such hypotheses.

The heterogeneity of GI pathology in infected and non-infected CVID patients suggests that gastrointestinal pathology may not always be directly related to specific types of infection or measured immunological parameters.

Several genetic diagnoses were made in our cohort which frame our findings. Histological colonic inflammation and, not at the time of biopsy, several infections with *Campylobacter* were seen in a patient with a *IKZF1* (encoding IKAROS) mutation. Cases of chronic diarrhea and IBD have been described in patients with *IKZF1* mutations [60]. Two patients with a CTLA4-insufficiency had colitis: IBD-like and lymphocytic colitis. This does not surprise, considering that about one-third of patients with reported CTLA4-insufficiency suffer from diarrhea, enteropathy, and IBD [61] and that CTLA4 is important in the regulation of intestinal inflammation [62]. There has been one report on a patient with coeliac-like disease and a *NFKB1* mutation [63] and on one patient with enteritis and giardiasis [64], which puts our findings of one patient with a coeliac-like histology into context. Whether patients with *NFKB1* mutations are even more susceptible to GI-infections will need to be further investigated. In patients with a mutation in *NFKB2*, giardiasis has been described previously, as well as gastrointestinal NLH [65], which was also seen in our *NFKB2*-patient, though without infection. *PTPN2* mutations have been described in patients with severe IBD [66] as it plays a role in balancing immune responses and preserving the intestinal barrier [67]. In our cohort, the affected patient had colonic inflammation, coeliac-like duodenitis, and NLH, was infected with norovirus, and had recurrent episodes of *Campylobacter* infections. *PTPN2* mutations are supposedly causing PID through an increased phosphorylation of STAT1 [68]. Even though the patient with a *SOCS1* variant [68] did not have histopathological abnormalities, this gene also plays a role in homeostasis of inflammation [69] and leads to an increased phosphorylization of STAT1. One patient with APDS had NLH and was positive for norovirus in a stool sample once. This is within expectation as lymphoproliferation is associated with APDS, as well as viral infections such as with herpesvirus [70]. Four patients had mutations in *TNFRSF13B* the gene encoding transmembrane activator and CAML interactor (TACI). These mutations (het (A181E), het(C104R), het(G278S)) have previously been published as disease-associated. All of them had gastrointestinal NLH. As described by Salzer et al. [71] and by Zhang et al. [72], and as observed in TACI knockout-mice [73], lymphoproliferation is a common feature in TACI-mutated patients and nodular lymphoid hyperplasia may occur in the gastrointestinal tract. The fact that four out of four of these patients have lymphoid hyperplasia in the gut is therefore expected. No signs of inflammation were seen in the reassessed biopsies.

“CVID enteropathy” in some studies has been defined as non-infectious, longstanding diarrhea, or, in other studies, as coeliac-like enteropathy. It is important that the scientific community comes to an agreement on which definition should be used. CVID enteropathy is an ambiguous term that causes misunderstandings and misinterpretations in clinical practice as well as in scientific research. We propose to use the term “CVID enteropathy” as a generic term for longstanding, non-infectious diarrhea with proven mucosal abnormalities in CVID patients as diarrhea in CVID may well be its own entity. However, our data suggest that there are recognizable subgroups within CVID patients with abdominal symptoms: coeliac-like enteropathy (generally associated with infection), inflammatory bowel disease with mucosal inflammation, NLH as the only feature, or even normal mucosa. Furthermore, genetic diagnoses might help to form groups as suggested by the results of our TACI-mutated patients who all had NLH. When histology and search for infections fail to explain a patient’s symptoms, other causes such as exocrine pancreatic insufficiency, small bowel bacterial overgrowth, intolerance to lactose or fructose, and irritable bowel syndrome should be considered.

A weakness of this study is that it is retrospective and not all data was produced at the same point of time. Information on reasons for endoscopy and possible infections were not universally available. Furthermore, histopathological features change depending on activity of disease and treatment, which could not be interrogated in this study.

## Conclusions

In summary, this study showed that the histological findings in CVID patients with diarrhea are very diverse, so that a single common cause is unlikely. Subgroups of histological changes in CVID patients with abdominal symptoms can be discriminated. However, testing for gastrointestinal infections should be performed routinely as they are associated with important histopathological changes and symptoms. In some cases, GI pathology is not related to specific infections and other related parameters seem to be hard to find. Biopsies were most often available of patients who had had endoscopy due to diarrhea, which makes conclusions on histology-symptom correlation harder. Biopsies should regularly be taken in CVID patients who undergo endoscopy, even if the procedure is performed for other reasons than diarrhea. Normal amounts of plasma cells may be present in the mucosal tissue of CVID patients, which is counterintuitive and should be further investigated. Carefully designed prospective studies are needed that reliably record the patient’s symptoms at the time of biopsy and an exhaustive infectious work-up at the time of biopsy is mandatory to be able to draw robust conclusions—not only in research but more importantly in every day clinical care of CVID patients.

## Supplementary Information

Below is the link to the electronic supplementary material.

Supplementary file1 (PDF 163 KB)

## Data Availability

All data generated or analyzed during this study are included in this published article (and its supplementary information files).

## Abbreviations

CMV: Cytomegalovirus
CVID: Common variable immunodeficiency
GI: Gastrointestinal
IBD: Inflammatory bowel disease
IEL: Intraepithelial lymphocytes
IvIg: Intravenous immunoglobulins
LH: Lymphoid hyperplasia
NLH: Nodular lymphoid hyperplasia
#: Number
PCs: Plasma cells
RFH: Royal Free Hospital London
