# Supplementary material for: Bowel Histology of CVID Patients Reveals Distinct Patterns of Mucosal Inflammation
Source: J Clin Immunol. 2021 Oct 1;42(1):46–59. doi: 10.1007/s10875-021-01104-5 (PMC8821476; doi:10.1007/s10875-021-01104-5)
Supplement: Supplementary file 1 — Supplementary file1 (PDF 163 KB) [file 10875_2021_1104_MOESM1_ESM.pdf]

Table S1: Descriptives of lymphocyte phenotyping

	N	Mean	Std.-Deviation	Minimum	Maximum
CD4+ first (*10^9/l)	44	0.6755	0.31873	0.22	1.72
CD4+ last (*10^9/l)	44	0.5807	0.36250	0.14	1.85
CD8+ first (*10^9/l)	44	0.6185	0.76801	0.07	5.16
CD8+ last (*10^9/l)	44	0.5852	0.97711	0.04	6.60
NK first (*10^9/l)	44	0.1644	0.10428	0.04	0.47
NK last (*10^9/l)	44	0.1478	0.11261	0.02	0.42
CD19+ first (*10^9/l)	23	10.4509	8.44200	0.12	32.97
CD19+ last (*10^9/l)	23	10.4943	8.36936	0.12	32.97
% of CD19 naive first	22	80.3623	12.85620	44.54	94.24
% of CD19 naive last	22	78.5068	11.56719	55.11	93.73
% of CD19 IgM memory first	22	10.4359	8.89329	1.92	39.87
% of CD19 IgM memorylast	22	11.5095	8.58444	3.34	39.87
% of switched memory cells first	22	3.8150	4.64308	0.20	20.64
% of switched memory cells last	22	3.9464	3.53630	0.20	13.47
CD3+ first (*10^9/l)	10	70.8675	26.97412	0.68	95.00
CD3+ last (*10^9/l)	10	70.0675	26.42371	0.68	95.00
% of CD4+ CD45RO+ CXCR5+ (TFH) (memory) first	11	18.000	10.5736	4.0	38.0
% of CD4+ CD45RO+ CXCR5+ (TFH) (memory) last	11	17.6364	10.79141	4.00	38.00
% of CD4 naive first	11	14.3636	11.94381	1.00	43.00
% of CD4 naive last	11	14.4545	11.87740	1.00	43.00
% of T-regulatory cells first	8	4.0000	2.00000	2.00	8.00
% of T-regulatory cells last	8	4.0000	2.00000	2.00	8.00
switched memory cells absolute first measured (*10^9/l)	22	0.4234	0.59068	0.01	2.46
switched memory cells absolute last measured (*10^9/l)	22	0.4076	0.43705	0.01	1.50
CD4+ naive absolute first measured (*10^9/l)	11	0.0940	0.11945	0.01	0.44
CD4+ naive absolute last measured (*10^9/l)	11	0.0853	0.09053	0.00	0.31

Table S2: Results of Mann-Whitney-U-Test to analyse the association of lymphocyte phenotyping results with histological features.

**Asymptotic Signifikance (2-sided) for**	coeliac-like	NLH only	IBD-like colitis	any colitis	Raised IEL	Lack of PCs	normal mucosa	villous atrophy	inflammation
CD4+ first (*10^9/l)	0.883	0.920	0.667	0.559	0.703	0.584	0.228	0.078	0.271
CD4+ last (*10^9/l)	0.285	0.496	0.763	0.515	0.603	0.245	0.560	0.808	0.544
CD8+ first (*10^9/l)	0.472	0.302	0.731	0.345	0.323	0.732	0.860	0.832	0.748
CD8+ last (*10^9/l)	0.210	0.313	0.931	0.686	0.253	0.891	0.989	0.585	0.647
NK first (*10^9/l)	0.238	0.267	0.931	0.261	0.368	0.657	0.881	0.362	0.674
NK last (*10^9/l)	0.113	0.496	0.796	0.486	0.253	0.356	0.989	0.281	0.720
CD19+ first (*10^9/l)	0.361	0.268	*	0.789	0.361	0.465	0.738	0.811	0.715
CD19+ last (*10^9/l)	0.361	0.356	*	0.285	0.361	0.465	0.640	0.811	0.927
% of CD19 naive first	0.472	0.491	*	0.885	0.472	0.886	0.647	0.688	0.819
% of CD19 naive last	0.632	0.533	*	0.664	0.632	0.534	0.647	0.841	0.424
% of CD19 IgM memory first	0.231	0.309	*	0.664	0.231	0.599	0.503	0.615	0.568
% of CD19 IgM memorylast	0.402	0.670	*	0.885	0.402	0.738	0.597	0.920	0.424
% of switched memory cells first	0.472	0.922	*	0.885	0.472	0.811	0.972	1.000	0.648
% of switched memory cells last	0.719	0.450	*	0.664	0.719	0.599	0.647	0.688	0.424
CD3+ first (*10^9/l)	0.192	0.909	0.513	0.248	0.192	*	0.602	0.569	0.917
CD3+ last (*10^9/l)	0.296	0.909	0.513	0.386	0.296	*	0.602	0.732	0.754
% of CD4+ CD45RO+ CXCR5+ (TFH) (memory) first	0.096	0.461	0.313	0.858	0.096	*	1.000	0.537	0.849
% of CD4+ CD45RO+ CXCR5+ (TFH) (memory) last	0.096	0.461	0.313	0.858	0.096	*	1.000	0.537	0.849
% of CD4 naive first	0.906	0.647	0.617	0.724	0.906	*	1.000	0.759	0.925
% of CD4 naive last	0.814	0.584	0.617	0.724	0.814	*	1.000	0.683	1.000
% of T-regulatory cells first	0.120	0.304	*	0.683	0.120	*	0.657	0.120	0.734
% of T-regulatory cells last	0.120	0.304	*	0.683	0.120	*	0.657	0.120	0.734
switched memory cells absolute first measured (*10^9/l)	0.632	0.974	*	0.885	0.632	0.811	0.698	0.763	0.819
switched memory cells absolute last measured (*10^9/l)	0.905	0.341	*	0.885	0.905	0.599	0.418	0.615	0.909
CD4+ naive absolute first measured (*10^9/l)	0.814	0.584	0.317	0.289	0.814	*	0.343	0.307	0.257
CD4+ naive absolute last measured (*10^9/l)	0.480	0.855	0.317	0.480	0.480	*	0.206	0.838	0.131

* Mann-Whitney-Test cannot be executed for empty groups.
